# Patient and clinician perspectives on the management of obesity in kidney failure prior to kidney transplantation: a mixed-methods systematic review

**DOI:** 10.1016/j.eclinm.2025.103649

**Published:** 2025-12-17

**Authors:** Zhanna Oganesova, Helen L. MacLaughlin, Kieran McCafferty, Sebastian Potthoff, Sharlene Greenwood, Victoria Vickerstaff, Rachel L. Batterham, Sarah A. Afuwape, Reza Motallebzadeh, Adrian Brown

**Affiliations:** a Centre for Obesity Research, University College London, London, UK; b Nuffield Department of Primary Care Health Sciences, University of Oxford, Oxford, UK; c School of Exercise & Nutrition Sciences, Queensland University of Technology, Brisbane, Australia; d Nutrition Research Collaborative, Royal Brisbane and Women's Hospital, Australia; e Queen Mary University and Barts Health NHS Trust, London, UK; f School of Communities and Education, Northumbria University Newcastle, UK; g Department of Renal Medicine, King's College Hospital NHS Trust, London, UK; h Renal Sciences, Faculty of Life Sciences and Medicine, King's College London, London, UK; i The Centre for Methodology and Evaluation, Queen Mary University of London, London, UK; j International Medical Affairs, Eli Lilly, Basingstoke, UK; k Department of Nephrology & Transplantation, Royal Free London NHS Foundation Trust, London, UK; l Department of Renal Medicine, University College London, London, UK; m Research Department of Surgical Biotechnology, Division of Surgery & Interventional Sciences, UCL, UK; n Bariatric Centre for Weight Management and Metabolic Surgery, University College London Hospital NHS Trust, London, UK; o National Institute of Health and Care Research, University College London Hospitals Biomedical Research Centre, London, UK

**Keywords:** Kidney failure, Obesity, Weight loss, Transplant, Dialysis

## Abstract

**Background:**

Obesity increases the risk of developing chronic kidney disease and progression to kidney failure (KF) and precludes kidney transplantation (KT). Challenges exist in people with KF losing weight to access KT, therefore understanding patients' and clinicians lived experiences of obesity management is crucial to improving equitable access to KT. This review aimed to synthesise qualitative and quantitative evidence to better understand patients' and clinicians’ experiences of obesity management in KF prior to transplantation.

**Methods:**

This mixed-methods systematic review followed the integrated methodological framework by the Joanna Briggs Institute. MEDLINE, Embase, and Web of Sciences were searched from 1st January 1980 to 16th April 2025 for studies investigating patients' and clinicians' perspectives on obesity management in KF. Qualitative, quantitative and mixed methods studies published in English in which patients or clinicians reported on their experiences of obesity management in kidney failure were included. Two investigators independently screened studies, extracted data, and assessed the risk of bias. Summary data were extracted from published reports and quantitative data underwent transformation into ‘qualitised’ data, Qualitative findings and qualitised survey results were analysed inductively using thematic synthesis. The study was registered with PROSPERO, CRD42024510237. The Mixed Methods Appraisal Tool version 2018 was used to evaluate the quality of selected studies.

**Findings:**

Of 6525 records identified, 5203 remained after de-duplication and 7 studies met inclusion criteria with a total of 738 participants The overall quality of the studies was low and only one study scored highly on the quality assessment. Four main themes were constructed 1) Hungry and exhausted: The impact of dialysis on eating behaviour and activity (six studies [n = 339]) 2) Weight stigma–lack of support, trust and open communication (five studies [n = 212]) 3) Lack of resources as a barrier for weight loss (six studies [n = 339]) 4) Who gets a transplant? Moving beyond BMI to improve equity in transplantation (four studies [n = 631]).

**Interpretation:**

Significant barriers to accessing and delivering obesity management were identified. When interpreting the results it should be appreciated that the overall quality of the studies was low. and clinician perspectives were limited to dietitians, nephrologists and transplant surgeons. To address these barriers, targeted strategies are recommended, such as enhanced training for health professional on obesity and communication about weight and weight stigma. There is an urgent paradigm shift needed to ensure equitable access to obesity management for people with obesity and KF.

**Funding:**

10.13039/501100000272National Institute for Health and Care Research.

**Research in context**

**Evidence before this study**

Obesity is an independent risk factor for chronic kidney disease and progression to kidney failure, affecting up to 30% of patients. A preliminary electronic search of databases MedLine, Embase and Web of Science was conducted from 1st January 1980 to 1st November 2023 scoping patients' and clinicians' experiences of obesity management in kidney failure before transplantation. Our search terms included “obesity”, “kidney failure”, “weight loss”, “patient”, “clinician” and “perspectives”. We identified several studies of either patients' or clinicians’ views of obesity management in kidney failure. There were no systematic reviews or meta-analyses identified as part of our preliminary search.

**Added value of this study**

To our knowledge, this is the first systematic review to synthesise patients' and clinicians’ perspectives on obesity management in kidney failure prior to transplantation. We constructed four key themes revealing significant barriers to both access and delivery in this population. Notably, our findings demonstrate that weight stigma is present in kidney services, undermining equitable access to weight management interventions and, ultimately, to transplantation itself.

**Implications of all the available evidence**

Our findings highlight the need for improved coordination of clinical roles, interprofessional collaboration, and greater patient social support. Urgent comprehensive obesity training for kidney clinicians is required alongside evidence-based weight management interventions. Furthermore, clinicians should be equipped to initiate discussions around weight with patients, recognise and address their own weight biases, while enhancing behavioural skills to support long-term weight loss. When interpreting the results it should be appreciated that the overall quality of the studies was low. and clinician perspectives were limited to dietitians, nephrologist and transplant surgeons. This study highlights the paramount need for specialised, multidisciplinary obesity management programmes to enable more equitable access for people living with obesity and kidney failure to kidney transplantation.

## Introduction

Obesity affects over 1 billion people globally^1^ and is an independent risk factor for chronic kidney disease (CKD), and progression to kidney failure (KF).^2^ The prevalence of people living with obesity (PLwO) and KF ranges between 6 and 30% across countries.^3^ Kidney Transplantation (KT) is the preferred treatment in KF due to better survival, quality of life, and lower costs compared to remaining on dialysis.^4^, ^5^, ^6^, ^7^ Yet, PLwO, especially women, experience lower rate of referral and listing for KT, along with longer waiting times, compared to people without obesity.^8^

Globally, many transplantation centres still require PLwO to lose weight to meet body mass index (BMI) thresholds for transplant eligibility, leading to exclusion of up to 30% of patients.^9^^,^^10^ Although obesity can increase risks of surgical complications after KT, including delayed graft function, incisional complications, and increased rates of graft loss, data indicates that PLwO who have received a transplant derive a significant survival advantage compared to those remaining on the waitlist.^11^, ^12^, ^13^ Yet, there remains limited evidence on the best metrics to predict obesity-related risks for KT and the most effective weight-loss strategies in PLwO and KF, particularly among those undergoing dialysis.^14^ This raises concerns, as PLwO may be denied transplantation based solely on weight, despite being otherwise eligible,^15^^,^^16^ and with limited effective, evidence-based weight loss strategies, particularly with those on dialysis, this increases KT inequity.

Achieving expeditious KT assessment and listing is crucial for long-term patient beneit, and therefore, addressing the challenges PLwO face accessing KT is an international priority.^17^^,^^18^ With the growing interest, and published guidelines in obesity management for people with CKD^19^ and KT candidates,^20^ understanding how patients and clinicians view obesity management in KF prior to transplantation is essential. This review examined patients' and clinicians’ experiences of obesity management in kidney failure before transplantation to highlight current practices, challenges, and gaps in obesity care for PLwO.

## Methods

### Search strategy and selection criteria

This review was formulated and undertaken in line with the convergent integrated methodological framework for mixed-methods systematic reviews proposed by the Joanna Briggs Institute (JBI).^21^ The protocol was prospectively registered on PROSPERO (CRD42024510237) and followed the Preferred Reporting for Systematic Reviews and Meta-Analysis Guidelines (PRISMA). An electronic search of databases: MedLine, Embase and Web of Science was conducted for papers published from 1st January 1980 to 16th April 2025. The search terms applied included “obesity”, “kidney failure”, “weight loss”, “patient”, “clinician” and “perspectives” (full Boolean search strings are provided in Supplementary Materials). Searchers were restricted to publications in English. Grey literature was not included given the focus on peer-reviewed evidence. The search was undertaken in two stages: an initial search covering 1 January 1980 to 19 January 2024, followed by an update search covering 20 January 2024 to 16 April 2025 alongside manual citation screening. The updated search identified one additional study that met the inclusion criteria.

The PICO framework^22^ was used to guide the selection criteria (Supplementary Material Table S1). No restrictions were imposed on the study design or sample sizes. Studies were assessed against the eligibility criteria utilising the screening software COVIDENCE where duplicates were removed then title and abstract screening, full-text review, data extraction and risk of bias were completed by two authors (ZO and AB), with cases of discordance resolved through iterative discussion until a consensus was reached.

### Data analysis

An integrated approach for mixed-methods reviews was employed for data extraction and transformation.^21^ The JBI mixed-methods data extraction form for reviews following a convergent integrated approach was used.^23^ Following data extraction, quantitative data underwent transformation into ‘qualitised’ data, involving narrative interpretation of the descriptive statistics from survey studies to directly respond to the research question.^21^ All text labelled as ‘findings’ or ‘results’ was considered, including participant quotations and primary researchers' interpretations. To avoid inflating the influence of larger surveys, no weighting by sample size or frequency counts was applied at the synthesis stage; instead, survey-derived narratives were entered as discrete textual findings each linked to its source study.

Thematic synthesis was employed to analyse qualitative and qualitised data to generate understanding and new overarching themes.^24^ All data were imported into Excel and coded and analysed using a thematic analysis framework.^25^ Coding was conducted inductively allowing themes to be identified directly from the data.^24^ Initially, ZO and AB independently performed line-by-line coding, followed by discussions to generate agreed codes and ensure intercoder agreement. The codes were then collated into preliminary themes which were further synthesised into descriptive summaries. The final themes were reviewed, refined and agreed through reiterative discussions, during which discrepancies were resolved, and clear descriptions for each theme were established.

The quality and risk of bias of the selected studies were assessed using the Mixed Methods Appraisal Tool (MMAT) version 2018: a critical appraisal tool designed for the appraisal of systematic mixed-method reviews.^26^

#### Positionality statement

We provide the following positionality details of the two authors involved data searching, extraction and synthesis to support readers in appraising how our identities and professional backgrounds may have influenced the conduct and interpretation of this review. At the time of writing, ZO self-identified as female and AB as male. Both authors self-identified as White. AB is a registered dietitian, and ZO is a researcher in primary care, both have professional experience in obesity management. Furthermore, AB is a weight stigma researcher with extensive clinical experience working with people living with obesity. Importantly, the wider author team contributed diverse perspectives across nephrology, transplantation, dietetics, behavioural science, and methodology, to ensure that the final themes were faithful to the data and resonant with clinical experience across specialties.

### Role of the funding source

The funder of the study had no role in study design, data collection, data analysis, data interpretation, or writing of the report. AB and ZO had full access to the data in the study, and all authors (ZO, HLM, KM, SP, SG, VV, RLB, SAA, RM and AB) had the final responsibility for the decision to submit for publication.

## Results

**Fig. 1:**
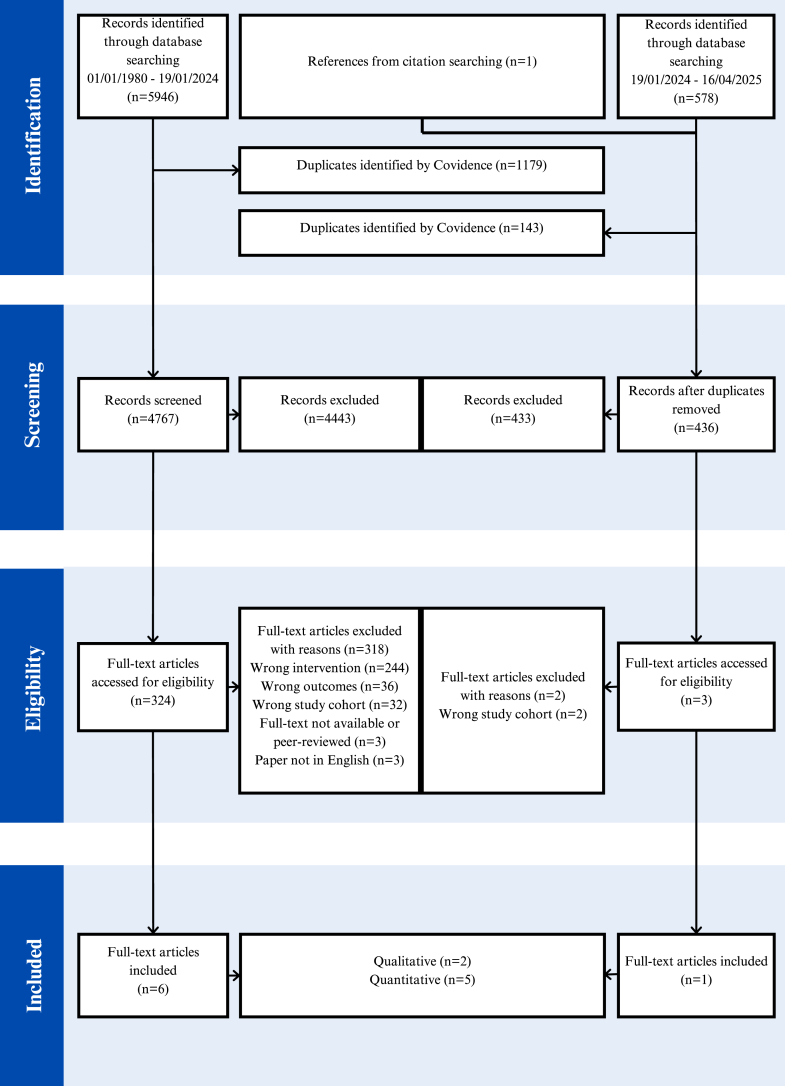
PRISMA Flowchart for identifying studies for systematic review. n, number of articles.

From a total of 6525 papers identified, 5203 papers were retrieved following duplicates removal. Seven studies were eligible (Fig. 1) including 738 participants with 243 patients (33%) and 495 clinicians (67%). The studies characteristics are shown for patients (Table 1) and clinicians (Table 2). Two studies were qualitative^27^^,^^28^ and five were quantitative.^29^, ^30^, ^31^, ^32^, ^33^ Four studies were conducted in the United States,^27^^,^^29^^,^^30^^,^^32^ one each in Denmark^28^ and Canada^31^ and one international.^33^ Three patient studies reported ethnicity, where patients were predominantly black ethnicity.^27^^,^^29^^,^^30^ Two studies reported patients attempting to lose weight including weight-loss surgery^27^ and other non-specified approaches.^29^ Patients' KT eligibility status was reported in one study, with most participants having undergone surgical assessment for KT.^30^ Four studies involved clinicians’ perspectives (n = 495) including dietitians (n = 41), nephrologists (n = 438), and transplant surgeons (n = 12).^27^^,^^31^, ^32^, ^33^

**Table 1:** Characteristics of the included studies with patients.

Author (year)	Country	Participants: Patients						Data collection/analysis approach	Outcome measure
		N (% Male)	Age (years)	BMI (kg/m^2^)	Ethnicity	Weight loss status	KT eligibility status		
**Qualitative studies**									
Harhay et al. (2023)^27^	United States	40 (42)	55 [46–63]	39.5 [35.3–41.6]	White–50% Black–35% Indigenous–2.5% Asia–2.5%	20% had undergone weight loss surgery	NS	Descriptive phenomenological approach Semi-structured interviews Qualitative deductive and inductive thematic analysis of interviews	Patients' lived experiences with obesity and weight-loss described as three themes
Freeman et al. (2021)^28^	Denmark	10 (70)	54 [42–66]	39.4 [29.3–51.2]	NS	90% losing weight for KT	Evaluated eligible −10% Not evaluated—90%	Descriptive phenomenological approach Semi structured interviews Qualitative inductive thematic analysis	Patients' experiences of obesity and weight-loss attempts described as four themes
**Quantitative studies**									
Saeed et al. (2017)^29^	United States	66 (46.5)	56 ± 13.8	33.5 [25.0–57.6]	White–21.2% Black–78.8%	23% had attempted weight loss	NS	12-question weight-related survey	Patients' opinions on weight-related issues including perceived health problems, reasons for desired weight-loss, barriers to weight-loss, and weight-loss strategies considered.
Gupta et al. (2019)^30^	United States	127 (48)	58 ± 16	27.3 ± 7.3	Black–100%	NS	Evaluated for eligibility–53% Evaluation in progress for eligibility −7%	Structured interview and survey	Association between patients' BMI and self-reported transplantation evaluation status, and perceived need for weight-loss

Note: Age and BMI expressed as either median [Inter-Quartile Range] or mean ± standard deviation; N, number, M, male; KT, kidney transplant; %, percentage; NS; not stated.

**Table 2:** Characteristics of the included studies with clinicians.

Author (year)	Country	Participants: Clinicians								Data collection/analysis approach	Outcome measure
		N (%M)	Age (Years)	RD %	N %	TS %	TD %	TN %	Years in profession		
**Qualitative studies**											
Harhay et al. (2023)^27^	United States	20 (30)	45 [39–52]	50	20	10	5	15	14 [8–22]	Descriptive phenomenological approach Semi-structured interviews Qualitative deductive and inductive thematic analysis of interviews	Clinicians' opinions on weight-related issues including perceived health problems, reasons for desired weight-loss, barriers to weight-loss, and weight-loss strategies considered
**Quantitative studies**											
Chan and Soucisse (2016)^31^	Canada	45 (NS)	NS	0	78	22	0	0	NS	18-item survey expressed as number and percentage	Standard descriptive statistics reflecting clinicians' experience in the assessment of candidates for transplantation, perceptions of the impact of morbid obesity on transplantation, views on bariatric surgery.
Suresh et al. (2020)^32^	United States	31 (NS)	<35 (22.6%) 35–50 (22.6%) 51+ (54.8%)	100	0	0	0	0	6 [5–14]	21-item survey and open-ended questions	Standard descriptive statistics reflecting clinicians' perceptions on the burden of obesity in KF, healthy weight-loss in dialysis settings, strategies, and challenges for obesity management.
Stenvinkel et al. (2013)^33^	Europe, South and Central America Middle East Asia North America Africa Oceania	399 (NS)	35–44 (19%) 45–54 (35%) 55–64 (30%) 12% not reported	0	100	0	0	0	NS	Eight question survey expressed as number and percentage	Standard descriptive statistics reflecting clinicians' knowledge and practice of managing obesity in the setting of CKD and ESRD.

Age and years in profession expressed as either median [Inter-Quartile Range] or percentage of participants.

TS, Transplant surgeon; TD, Transplant dietitian; TN, Transplant nephrologist; N, Nephrologist; RD, renal dietitian; %, percentage; M, males; NS, not stated; KF, kidney failure; CKD, chronic kidney disease; ESRD, end-stage renal disease.

### Assessing methodological limitations of included studies

The MMAT appraisal revealed the overall quality of the studies was low, apart from one study which was assesses as high quality.^27^ The other included studies had substantial methodological limitations including unclear research questions,^28^ the use of non-probability sampling with inadequate reporting of strategies, population descriptions, and response rates, resulting in high risk of sampling and nonresponse bias except Gupta et al., 2019.^30^ Furthermore, the survey-based data collection lacked pre-testing and reliability checks, increasing measurement error; and statistical analysis was insufficiently reported in most studies, with only Saeed et al. (2017)^29^ providing comprehensive methods (full appraisal in Supplementary Table S2).

### Synthesis

Four main themes were constructed, with associated sub-themes. Theme summaries and descriptions are described in Table 3. Partial and full quotes are used to illustrate points, with full representative sample quotes being presenting in Table 4.

**Table 3:** Description of four key themes and subthemes.

Theme	Definitions	Sub-theme	References
Hungry and exhausted: The impact of dialysis on eating behaviour and activity	This theme revealed diet and exercise as the most common obesity management approaches in kidney failure. Patients reported difficulties following weight loss advice due to dietary and fluid restrictions. Furthermore patients expressed challenges associated with dialysis treatment such as hunger and exhaustion increasing the difficulty of making changes to diet and exercise.	Balancing weight loss and kidney failure dietary advice	^27^^,^^28^^,^^30^^,^^32^
		Exhaustion as a barrier to exercising	27, 28, 29, 30, 31, 32
Weight stigma–lack of support, trust and open communication	This theme revealed the challenges patients face in navigating the power dynamics and communication barriers with clinicians regarding weight management. It reflected how potential clinician biases lead to inadequate support and advice, while patients grapple with feelings of responsibility and self-blame regarding their weight and being unable to make changes to their lifestyle.	Discussing weight: Clinician bias as a barrier to open communication	27, 28, 29^,^^31^^,^^32^
		Internalised weight bias and the ‘weight’ of responsibility	^27^^,^^28^

Theme	Definitions	Sub-theme	**Source**
Lack of resources as a barrier to weight loss	This theme revealed lack of time and obesity knowledge as key barriers that hindering both patients and clinicians from addressing obesity effectively. While clinicians lacked adequate obesity training, patients struggled with financial aspects of following a healthy diet to help weight loss.	When is the right time?	27, 28, 29^,^^31^^,^^32^
		It's not my job	^27^^,^^32^
		Food insecurity	^27^^,^^29^^,^^32^
		Obesity–Helps or Hinders? Role of knowledge and experience in guiding weight loss decisions	^27^^,^^29^, ^30^, ^31^, ^32^
Who gets a transplant? Moving beyond BMI to improve equity in transplantation	This theme revealed the lack of consistency and equity in evaluations of kidney transplant candidates. While BMI was used by clinicians as the key proxy in assessments, patients argued that it inadequately reflected overall health, leading to frustration over being denied evaluation for transplant based solely on their overall body size.		^27^^,^^30^^,^^31^^,^^33^

Abbreviation: BMI, Body mass index.

**Table 4:** Example of full participant quotes with contextual explanation to frame the quote.

Quote	Contextual explanation	Entire quote
1	Here this patient highlights these challenges of weight loss	*‘I have tried to eat reasonably, but I find it difficult since there are so many things I can't eat’.*^28^
2	Here this patient explained how they felt after dialysis regarding their hunger and explaining that a doctor had told them what?	*‘after dialysis, usually you're famished. If I don't eat something, then I know it can suck more energy out of me, because your body needs something.’*^27^
3	Here this patient expressed they would need to cope with exhaustion before incorporating exercise	*‘I need to find a way to deal with the exhaustion, that's something I need to find a rhythm in before I can add exercise to my schedule’.*^28^
4	Here this patient explains the need for support to engage with activity	*‘I would like to meet with someone and exercise. If I have to go to the gym alone, I just won't get it done’.*^28^
5	Here this clinician remarked about not discussing weight with a patient	*‘Rarely do any of [the patients] really tell me, ‘Oh, I'm overweight,’ or ‘I'm obese, and I would like to lose weight’.*^27^
6	Here is dietitian expresses they have too much other work to be able to focus on weight loss	*‘We have so much other stuff to focus on that obesity and weight management is not always the top priority unless the patient makes it a priority’.*^27^
7	Here this clinician describes their opinions related to patients following diet advice	*‘[Patients] claim to not be eating anything at all … and then their phosphorus is through the roof. … I think it's a lot of lying’.*^27^
8	Here this clinician described the need for lose weight in order to get a transplant	*‘And in order to prove that you're a good steward of the organ transplant when we have this obesity, it really helps to see that the patient is engaged enough to lose weight’*^27^
9	This patient explains the amount of weight to be listed for transplant as too much and they would not be able to achieve it	*‘They told me to lose*10 kg*, and my initial reaction was that I couldn't, and that it wasn't going to happen’.*^28^
10	Here this HCP explains their belief about the benefit of targets	*‘[Having a BMI barrier for transplant] was helpful in terms of care. There were two or three patients that were able to lose significant amount of weight. … [Now] we have a transplant-surgery group now that has removed the BMI criteria … [and] the motivation for weight loss is not quite what it was prior to that.’*^27^
11	Here this patient expresses their frustration in not being able to lose weight	*‘I'm so annoyed that I can't just do it, I think to myself—why don't you just do it?!’*^27^
12	Here this patient expressed the challenges of waiting to lose weight	*‘It wasn't until I started in dialysis that it became serious to me, when you suddenly can't do anything at all, it's time to get in gear.’**^29^*
13	Here a transplant clinician expressed their thoughts on who should help patients with weight loss	*‘I think because dialysis dietitians are seeing their patients so much more frequently … it may even be more beneficial for a dialysis dietitian to be more involved in the weight loss aspect of things.’*^27^
14	Here this clinician explains their reluctance to advise bariatric surgery to a patient	*‘I'm not sure I would feel real good about advising someone to go for bariatric surgery to lose weight to go for a transplant because it's a lot of surgery in the abdomen.’*^27^
15	Here another clinician expresses their reservations about gastric bypass surgery	*‘We don't ever recommend somebody to get a gastric bypass so they can lose weight for a transplant. I don't even recommend gastric bypass at all for any person.’*^27^

### Theme 1: hungry and exhausted: the impact of dialysis on eating behaviour and activity

This theme appeared in six papers^27^, ^28^, ^29^, ^30^, ^31^, ^32^ revealing diet and exercise were the most common weight-loss interventions in patients with KF. Though patients reported challenges in following them due to dialysis-related dietary and fluid restrictions, hunger and exhaustion.

#### Subtheme (ST) 1: balancing weight loss and kidney failure dietary advice

Dietary advice, including portion control, mindful eating and self-monitoring, was the most common obesity management intervention in KF.^27^, ^28^, ^29^, ^30^, ^31^, ^32^ However, it was *‘extremely hard’*^28^ for patients to adhere due to *‘incompatible*’^27^ dietary restrictions, hunger and cravings. Patients reported frustration, struggling to lose weight as advice for weight loss conflicted with dietary restrictions for KF^27^^,^^28^^,^^32^ (Quotation 1, Table 4).

Maintaining an energy deficit was reported as challenging while balancing potassium and phosphate recommendations, alongside fluid allowances.^27^^,^^28^ This was described as a ‘*fine line’*^27^ requiring being ‘*careful all the time’*.^28^

Dialysis itself was a substantial barrier to weight loss as it impacted patients' eating behaviour, energy and hunger (Quotation 2, Table 4).^27^ Post-dialysis fatigue affected their ability to prepare healthy meals, leading patients to consume fast or processed foods.^27^ Eating was also used as a coping mechanism to manage stress and boredom during dialysis, *‘I'm so bored, I need something’*.^27^

#### ST 2: exhaustion as a barrier to exercising

Exercise was the second most common obesity management approach in KF, but patients found it difficult due to the exhaustion from dialysis.^28^, ^29^, ^30^, ^31^, ^32^ Despite recognising its benefits,^28^ dialysis frequency, work commitments and living with obesity itself, left patients with ‘*too little energy*’ [fatigue] for exercise^28^ (Quotation 3 and 4, Table 4).

### Theme 2: weight stigma—lack of support, trust and open communication

This theme appeared in five papers,^27^, ^28^, ^29^^,^^31^^,^^32^ capturing communication barriers between patients and clinicians. It reflected how clinician bias appeared to lead to inadequate support and advice, while patients contended with feelings of responsibility and self-blame.

#### ST 1: discussing weight: clinician bias as a barrier to open communication

Clinicians rarely initiated conversations about obesity and weight*’*^27^ sometimes due to not wanting to ‘*embarrass them [patients]’*^27^ while others identified the clinical environment [dialysis] as a barrier to discussing weight loss due to there being “*not much privacy [at the dialysis facility]*”^27^ or that *“other people can hear”*.^27^

Clinicians expected patients to recognise obesity as an issue themselves, and initiate conversations about wanting to address it,^27^ though patients rarely did (Quotation 5, Table 4). Clinicians also reported other clinical workload as more important (Quotation 6, Table 4). This sometimes contrasted with patients’ views, with several reporting being interested in losing weight,^27^ while others felt clinicians discussed everything but weight.^27^

When discussing obesity with patients, clinicians confined it ‘*to the importance of achieving a goal BMI for transplant eligibility*’,^27^ though some did not recommend weight loss unless for transplantation.^32^ Simultaneously, meeting BMI requirements for KT was not always the patients' main goal.^27^^,^^28^ Some expressed wanting to lose weight to improve mobility, decrease pain, and experience less discrimination,^27^^,^^29^ with others feeling weight loss was ‘*the only choice*’^28^ to improve their health.

Although patients' views on HCPs support varied, they appeared frustrated the advice ‘*did not suit them*’.^27^^,^^28^ Despite advice sometimes being *‘good’*, it was also perceived as ‘*the same old song*’,^28^ lacking understanding for the challenges they faced and was ‘*very hard to follow*’.^28^ Clinicians were described as focusing on nutritional status, leaving some patients feeling ‘*overly scrutinised*’ and receiving ‘*little culturally concordant or holistic*’^27^ weight-loss advice. One patient described feeling ‘*pushed*’ into bariatric surgery to achieve transplant listing yet received no dietary support.^27^ Patients also expressed not wanting to be *‘caught doing something bad’,* linking their struggles with dietary advice to a sense of being *‘the kid in me’*.^27^

Of concern was clinicians' apparent bias and negative attitudes towards PLwO. They reported having little trust in patients, describing them as ‘*childlike*’, *‘lying’*, and unable to adhere to the dietary guidance^27^ (Quotation 7, Table 4). Furthermore, patients' compliance with weight loss was at times perceived as a proof of ‘*good stewardship’* for transplant eligibility^27^^,^^31^ (Quotation 8, Table 4).

#### ST2: internalised weight bias and the weight of responsibility

Although patients were frustrated by the lack of obesity management support, several believed weight loss was ultimately their responsibility, saying ‘*no one else could do the job for them*’.^27^ Patients commented on being mentally exhausted and feeling hopelessness.^28^ The emotional toll of managing obesity was overwhelming when faced with the amount of weight patients were advised to lose^28^ (Quotation 9, Table 4). The “unrealistic” goals undermined motivation to even initiate weight loss, as patients doubted they were achievable.^28^ Contrastingly, several clinicians suggested BMI and weight targets being useful in motivating weight loss^27^ (Quotation 10, Table 4).

Patients described weight loss as a ‘*battle*’ and ‘*entirely impossible*’,^28^ expressing frustration with their inability to lose weight despite their efforts, leading to self-blame and guilt^27^ (Quotation 11, Table 4). Some struggled to engage with advice and wanted someone who could ‘*keep an eye*’ on them and help with accountability.^28^ Meanwhile, a patient who lost weight attributed their success solely to “*self-discipline and nothing else*”.^27^

### Theme 3: lack of resources as a barrier for weight loss

This theme appeared in six papers,^27^, ^28^, ^29^, ^30^, ^31^, ^32^ revealing resource-related barriers such as lack of time, limited obesity knowledge, and funding that reduced both patients' and clinicians’ ability and motivation to address obesity.

#### ST 1: when is the right time?

Lack of time was important in determining patients' sense of urgency and clinicians' priority for obesity management in KF.^28^^,^^29^^,^^32^ Patients needed additional ‘*room*’ and ‘*extra time*’ to ‘*take on the weight loss battle*’.^28^ Some did not feel urgency to lose weight until their CKD became more severe,^28^^,^^29^ though acknowledged waiting could impact their chances of weight loss (Quotation 12, Table 4). Notably, both patients and clinicians reported a lack of time and motivation to actively pursue or support weight loss,^29^^,^^32^ yet, setting a time frame for patients to achieve weight-loss goals was uncommon.^31^^,^^32^

#### ST 2: It's not my job

A lack of obesity training was prevalent among clinicians.^27^^,^^32^ Nephrologists and renal dietitians expressed not being ‘*adequately trained in obesity management*’,^32^ and it was ‘*outside their scope of practice*’.^27^ They acknowledged the importance of obesity education but suggested that a ‘*weight loss expert*’^27^ should instead be available for patients wishing to lose weight. Clinicians were unsure about their roles in addressing obesity in dialysis^27^ and identified the absence of obesity management guidelines as a key challenge.^32^

Concurrent renal workload was a key barrier in supporting weight loss in dialysis.^32^ Renal dietitians reported ‘*feeling inundated*’^27^ with dialysis-related care and administrative tasks and could not prioritise weight management.^27^ Several felt dialysis was not the right time for delivering ‘[dietary] *education’*, citing patients' ‘*disoriented thinking*’ and ‘*altered mental status*’ during dialysis as an issue.^27^ Transplant clinicians emphasised the importance of rapport, viewing dialysis dietitians as best placed to provide advice due to their frequent patient contact (Quotation 13, Table 4).

#### ST 3: food insecurity

Both patients and clinicians identified ‘*food access*’ [ability for patients to afford food],^32^ particularly cost and inaccessibility, as a major barrier to weight loss in KF.^27^^,^^29^ Patients struggled to afford ‘healthy foods’, while clinicians, including renal dietitians, stressed the need for *‘education on healthy food choices despite food insecurity’*,^32^ suggesting monitoring food access as part of obesity management.^25^

#### ST 4: obesity–helps or hinders? Role of knowledge and experience in guiding weight loss decisions

Many patients were interested in losing weight, although goals varied,^29^ with some aiming at maintenance, while one wishing to gain weight, despite living with obesity.^30^ Patients cited both cosmetic and health-related reasons for maintaining a weight higher than recommended.^27^ Some associated thinness with being ‘*frail or sick*’, with patients expressing that; *‘Judging from what I see in other dialysis patients, skinny people die quicker’*.^27^

Notably, patients tended to underestimate their own weight and not consider themselves to be living with obesity,^29^ with years of education and awareness of transplantation centre weight limit being associated with trying to lose weight.^30^ Patients themselves reported the lack of knowledge as a barrier to weight loss and considered obesity education important.^29^

Lack of obesity knowledge was also prevalent among clinicians.^27^^,^^32^ Clinicians expressed differing and sometimes contradicting perspectives on the need and safety of weight loss on dialysis.^27^^,^^31^ Similarly to patients, some believed obesity was protective, allowing for better survival on dialysis, with this HCP saying *‘Obese people tend to live longer on dialysis. They survive longer on dialysis’*.^27^ Attempting weight loss was sometimes seen as ‘*futile*’^31^ and ‘*associated with too much risk*’^31^ and could impact on nutritional status, especially protein depletion,^31^^,^^32^ with some advising increasing protein intake to mitigate risk.^32^

Clinicians spoke about the importance of *‘healthy weight loss’*, which may require changes to the standard monitoring of PLwO and KF.^32^ This included looking for *‘temporal and interdigital wasting, arm and leg muscle wasting’*, and *‘hair loss, and slow healing’*,^32^ which may not have been standard practice for PLwO. Weight loss was primarily monitored through *‘Diet recalls’* and *‘lab results’,* with *‘social support’* also considered,^32^ with monthly weight losses of less than 5% encouraged.^32^

Most clinicians felt patients should lose weight before KT, with some suggesting they should lose all excess weight before listing,^31^ though ‘excess weight’ was not defined. Many believed obesity increased risks of ‘*intra-operative and post-transplant complications’*^31^*,* decreasing patient and graft survival. Notwithstanding, there was no consensus on how best to achieve weight loss, with varied perspectives on surgical and pharmacological interventions among both patients and clinicians.^27^^,^^29^^,^^31^ Generally, clinicians' experience with bariatric surgery was limited.^31^ Nephrologists, surgeons, and transplant nephrologists appeared relatively enthusiastic regarding bariatric surgery prior to and while on dialysis, or for KT access.^27^^,^^31^ Clinicians reported having ‘*evidence-based*’ conversations about the benefit of bariatric surgery and highlighted the need for long-term data.^27^ Contrastingly, others described bariatric surgery as too invasive and hesitated to recommend it (Quotation 14 and 15, Table 4). Concerns included post-operative bariatric complications such as nephrolithiasis, anastomotic leaks,^31^ and the potential impact on immunosuppressive medication absorption.^31^ A BMI of 35 kg/m^2^ and 40 kg/m^2^ were primarily used as thresholds for bariatric surgery referrals^31^ which often occurred prior to listing or referral for KT.^31^

Similarly, patients had limited experience or interest in bariatric surgery.^27^^,^^29^ Some, like clinicians, viewed it too invasive and ineffective without a *‘change in eating habits’*.^27^ Concerns regarding *‘weird side effects’* reduced patients' enthusiasm, with some patients believing it only *‘helps some’*, while others may *‘use it as a crutch’*.^27^ One patient, however, described bariatric surgery as life-changing and expressing regret for not pursuing it earlier to avoid ‘*years of pain*’ they experienced.^27^

Side-effects were also a concern for patients taking obesity management medications (OMMs). One patient discontinued treatment due to appetite suppression, saying it made them ‘*not want to eat at all’*.^27^ OMMs were rarely considered by clinicians to manage obesity in KF, potentially due to limited experience using them.^27^^,^^29^

### Theme 4: who gets a transplant? Moving beyond BMI to improve equity in transplantation

This theme appeared in four papers revealing clinicians considering obesity during transplant eligibility assessments but describing centre-level inconsistency in local obesity policies.^27^^,^^30^^,^^31^^,^^33^ There was no consensus on how to measure body composition or operationalise eligibility for KT.^31^^,^^33^ Most clinicians believed morbid obesity^31^ was a contraindication for KT, with half suggesting a BMI 40 kg/m^2^ as an appropriate limit, while others advocated for 30–35 kg/m^2^.^31^^,^^33^ Some clinicians were concerned that BMI limits unfairly excluded patients.^31^

PLwO who were denied assessment for transplantation expressed frustration, arguing it was *‘wrong’* and *‘unfair’* to evaluate eligibility by a single number.^27^ They viewed BMI as a poor proxy for health, reporting that they felt they had to wait until they were *‘half-dead’* to be considered. Patients advocated alternatives such as waist circumference and broader appraisal of body habitus.^27^ In agreement, some clinicians reported using abdominal circumference, fat distribution, and compliance when assessing patients for transplantation.^31^

## Discussion

This review is the first to synthesise patients' and clinicians’ perspectives on obesity management in KF prior to transplantation. Multiple patient challenges were revealed in achieving weight loss but also from the clinicians in supporting it.^34^

Obesity is a complex, relapsing, and progressive condition^35^ requiring tailored interventions with timely escalation based on individual need.^36^ PLwO face various barriers to weight loss including lack of support, time constraints and emotional strain.^28^ For PLwO and KF, these challenges were also present but compounded by dietary and fluid restrictions, alongside exhaustion and hunger from dialysis, making weight loss even more challenging. Despite this, diet and exercise remained the most common weight-loss interventions, which were rarely adapted to appreciate these challenges, potentially limiting their effectiveness.

Simultaneously, there were polarised views among HCPs regarding more intensive interventions such as bariatric surgery and OMMs, with some clinicians actively avoiding suggesting these interventions. This reluctance appeared partially influenced by their limited experience, lack of access to effective interventions and concerns about potential post-bariatric complications, and impact on immunosuppressive medication. However, some reasons appeared more arbitrary, for instance, one clinician explained, ‘*it's a lot of surgery in the abdomen’,* suggesting at times opinion, rather than evidence-based guidance, shaped clinical decisions. Similarly, referral decisions for transplantation appeared to not always based on objective clinical criteria but hinged on subjective opinion such as patients being a ‘*good steward of the organ transplant’* if they lost weight. Such moralisation, where the ability to lose weight is framed as a proxy for self-discipline,^37^ and thus, worthiness, raises concerns about fairness and consistency in clinical decision-making. This may reflect underlying weight stigma among kidney HCPs, risking exacerbating inequitable KT access.

Weight stigma is highly prevalent within society and especially healthcare,^38^, ^39^, ^40^ impacting directly on the care PLwO receive.^38^ Currently, despite obesity kidney guidelines acknowledging weight stigma^19^ there is a dearth of research in CKD and KF. Internalised weight bias was apparent among patients’ comments, manifesting in self-blame, guilt, and weight loss being solely their responsibility. With internalised weight stigma negatively impacting both psychological and physical health,^38^^,^^41^ there is a need for HCPs to help support patients to recognise and address these feelings.

Concerningly weight-biased attitudes were present among kidney HCPs. Several clinicians described patients as *‘child-like’*, unmotivated, and unable to adhere, with some expressing patients were *‘lying’* about what they ate.^27^ Such sentiments reflect wider societal weight bias, where PLwO are stereotyped as lazy, dishonest, and weak-willed.^38^^,^^42^^,^^43^ This dynamic appeared to make patients feel infantilised, and afraid of being ‘*caught’* when eating foods they think they should not.^27^ Such ‘*adult-child*’ relationships are problematic, as it undermines the mutuality of participatory power and decisional capacity of both parties.^44^ Clinicians' mistrust alongside patient fear of punishment potentially fosters a climate of suspicion and care disengagement. Therefore specialised training should be implemented to raise clinicians' awareness and capacity to reflect on weight bias and how this impacts patient care.^45^

Both patients and clinicians reported low motivation and diminished prioritisation to address obesity. Our data suggested stress, fatigue and time constraints faced by both patients and clinicians,^46^ reduced reflective motivation by making behaviour change less appealing and by depleting the self-regulatory resources required to sustain these. Quotes from HCPs reflected uncertainty, with dialysis and transplant teams suggesting obesity was not their job. This deflection appeared to lead to clinical inertia, where ambiguity around roles resulted in obesity being deprioritised. It is therefore, critical for kidney teams to clarify clinical responsibilities for obesity management. Organisational constraints, such as clinical workload, limited time, where care is delivered, and lack of training, must also be addressed to improve obesity care and reduce potential clinical inertia. This inertia to address obesity appeared partially related to an absence of renal guidelines on obesity management at the time of these studies. The recent guidance from the American Society of Nephrology^11^ on managing obesity in people with CKD provides a renewed focus, highlighting available tools and potentially increasing clinicians’ confidence in treating PLwO.

Importantly, knowledge gaps were evident among both patients and clinicians and could play a role in precluding obesity management in KF.^47^ Clinician narratives indicated uncertainty and inconsistency regarding whether, when. and how to pursue weight loss in KF.^19^^,^^20^ Notably, some HCPs and patients commented that obesity was protective in dialysis,^48^ though it was not entirely clear if this was prohibitive of advising or initiating weight loss, respectively. This appeared to refer to the so-called obesity-paradox, where obesity is paradoxically associated with better survival in patients on haemodialysis.^48^^,^^49^ However, controversy remains, with criticism of this association being a consequence of methodological limitations including collider stratification and misclassification bias and reverse causation,^20^^,^^50^ therefore a clearer understanding of the relationship between BMI and survival is needed. Furthermore, there was no consensus on BMI thresholds or use of other criteria based on body fat distribution, especially for those with *‘morbid obesity’*.^20^^,^^31^^,^^33^^,^^51^^,^^52^ Notwithstanding, patients were expected to meet, what they perceived as an ‘unfair’ and arbitrary, BMI target for transplantation,^27^ expressing frustration with not understanding the reasons for not being listed or assessed. Contrastingly, HCPs suggested BMI cut-offs as motivating which clearly contradicted with the hopelessness expressed by patients to lose weight.

Indicatively, communication emerged as a key barrier, both with clinicians initiating weight discussions and patients themselves broaching the topic with HCPs. The challenges clinicians face in bring up weight have been extensively studied with lack of time, training and concerns about causing offence being identified,^53^, ^54^, ^55^, ^56^ which were consistent with our findings. A unique challenge identified in KF was the dialysis environment itself, with clinicians expressing dialysis was an inappropriate time to discuss weight due to concerns over privacy, causing embarrassment and impact of so called *“dialysis fog”* (the acute impact of dialysis on cognition and memory).^57^^,^^58^ Thus in attempting to avoid causing distress, clinicians failed to offer support.^58^ Patients however appeared to want to discuss weight loss, though at the same time, avoided the conversation themselves, which may have been driven by previous experiences of weight stigma,^59^ resulting in inaction by both parties. To address communication issues, training is required to equip kidney clinicians to confidently bring up the conversation of weight in a sensitive and appropriate manner.

Patients reported a desire for greater social support, highlighting its value in fostering motivation and accountability.^28^ Given social support has been associated with improved satisfaction, adherence, and even survival in patients on haemodialysis,^60^^,^^61^ improving social support in obesity management and not only focus on kidney specific issues appears essential. This knowledge could help in designing support systems that foster healthy habits through consistent social cues and positive reinforcement in line with behavioural theories^46^ enabling more sustainable weight loss.

This review has several strengths. This is the first review synthesising both patients' and clinicians’ opinions and offers important perspectives regarding obesity management in KF. The methodology allowed inclusion of both quantitative and qualitative studies enabling greater insights on a topic with limited research. Finally, this is one of the first studies to identify that weight stigma may be impacting access to weight loss interventions and transplantation and therefore warrants further research.

These review findings should also be interpreted with several limitations in mind. The overall quality of the studies was low, and several studies over five years old^29^, ^30^, ^31^^,^^33^ which may not reflect current practices in obesity management in KF. Clinician perspectives were largely limited to renal dietitians, nephrologists, and transplant surgeons, with minimal input from other multidisciplinary team members such as nurses, psychologists, and physiotherapists. Additionally, most studies^27^^,^^29^, ^30^, ^31^, ^32^ were conducted in North America, limiting generalisability of the findings to other countries. Finally, consideration should be taken to the potential temporal shifts in clinical perspectives, given the publication dates of the included studies were over a decade and the impact of evolving clinical practice and patient views.

In conclusion, despite mounting international interest in obesity management in KF, which may be in part being driven by the new OMMs in earlier stages of CKD^62^^,^^63^ and their potential use in people with KF, there remains a lack of qualitative research in the area. This systematic review synthesised current evidence of patients' and clinicians’ perspective on obesity management in KF prior transplantation, highlighting the pressing need for a paradigm shift in practice. This review highlights the complexities and systemic barriers in managing obesity in patients with KF, including the lack of obesity-related clinician training, the absence of specialised renal obesity services, and inadequate patient-clinician communication. Furthermore, concerns were raised about the potential impact of weight stigma on clinical decisions.

To address these issues, improved coordination of clinical roles, interprofessional collaboration, and greater patient social support are essential. Comprehensive obesity training for kidney clinicians is urgently required, focusing on the science of obesity, its interplay with KF, and evidence-based weight management interventions.^39^ Furthermore, clinicians should also be equipped to initiate discussions around weight with patients and recognise and address their own weight biases, while honing skills in behavioural counselling to support long-term weight loss. Given the complexities of managing obesity in KF, there is a paramount need for specialised, multidisciplinary obesity management programmes to enable more equitable access for PLwO and KF to kidney transplantation.

## Contributors

AB contributed to conceptualisation, data curation, formal analysis, funding acquisition, investigation, methodology, project administration, supervision, validation, writing—original draft, and writing—review & editing. ZO contributed to data curation, formal analysis, investigation, methodology, validation, writing—original draft, and writing—review & editing. AB and ZO had access to and verified the underlying study data. HLM formal analysis and writing—review & editing. SP, SG, VV, RLB, SAA, RM contributed to writing—review & editing. All authors gave final approval of the manuscript.

## Data sharing statement

All thematic theme data generated is included in this published article and its Supplementary Information Files, alongside search terms and methodology. The extracted data and quotes used in this systematic review have been deposited in the figshare repository and are accessible via the following https://doi.org/10.6084/m9.figshare.29614013.

## Declaration of interests

AB declares researcher-led grants from the National Institute for Health Research, Rosetrees Trust, MRC, INNOVATE UK, British Dietetic Association, British Association of Parenteral and Enteral Nutrition, BBRSC, the Office of Health Improvement and Disparities and NovoNordisk. AB reports honoraria from Novo Nordisk, Mac Nutrition, Cordon Bleu and Eli Lilly outside the submitted work and is on the Medical Advisory Board and shareholder of Reset Health Clinics Ltd. RB declares researcher-led grants from the National Institute for Health Research, Rosetrees Trust, Sir Jules Thorn Biomedical Trust and NovoNordisk. RB reports honoraria from Novo Nordisk, Eli Lilly, Medscape, ViiV Healthcare and International Medical Press and advisory board and consultancy work for Novo Nordisk, Eli Lilly, Pfizer, Gila Therapeutics, Epitomee Medical and ViiV Healthcare and from May 2023 is an employee and shareholder of Eli Lilly and Company. HM declare researcher-led grants from Queensland Health Office of the Chief Allied Health Officer 2023–2025. HM reports support from KDIGO supporting travel to attend conference and is Deputy Chair Chronic Kidney Disease Work Group: Australasian Clinical Trials Network. RM declares research-led grants from the National Institute for Health Research, Kidney Research UK, Rosetress Trust and Royal Free Chrity. SG is director of Kidney Beam Ltd. SAA is on the trial management group of fellowship for AB. ZO, KM, SP, VV, report no conflicts of interest.
