# Supplementary material for: Patient and clinician perspectives on the management of obesity in kidney failure prior to kidney transplantation: a mixed-methods systematic review
Source: eClinicalMedicine. 2025 Dec 17;91:103649. doi: 10.1016/j.eclinm.2025.103649 (PMC12770947; doi:10.1016/j.eclinm.2025.103649)
Supplement: Supplementary Material [file mmc1.pdf]

Supplementary Material

The Boolean string used for investigating patient and clinician perspectives on obesity management in kidney failure prior transplantation.

(Obes* OR overweight OR adipo* OR body mass OR weight* OR BMI) AND (intervention OR manag* OR approach OR treatment OR therap* OR surg* OR diet* OR nutrition* OR food ADJ3 intake OR eating OR meal OR calor* ADJ3 restrict* OR feeding OR exercis* OR physical ADJ3 activity OR training OR fitness OR active ADJ3 lifestyle OR sport* OR bariatric OR gastric ADJ3 surgery OR weight ADJ3 reduction OR laparoscopic ADJ3 surgery OR sleeve gastrectomy OR gastric bypass OR gastric sleeve OR gastric banding OR gastric restrictive surgery OR intragastric surgery OR gastroplasty OR semaglutide OR glucagon-like peptide receptor agonists OR GLP-1RAs OR ozempic OR SGLT2 inhibitors OR liraglutide OR naltrexone OR bupropion OR orlistat OR phentermine OR pharmacological OR weight* ADJ3 medic* OR weight* ADJ3 drug*) AND (Hemodialysis OR haemodialysis OR dialysis) AND (kidney ADJ3 failure OR end stage kidney disease OR end stage renal disease OR kidney ADJ3 injury OR renal failure) AND (patient* OR person* OR recipient* OR individual* OR people) AND (experien* OR expect* OR challeng* OR eligib* OR view* OR attitude* OR motivat* OR perspective* OR feedback OR insight* OR report* OR reflect* OR account OR feeling* OR qualitative OR thematic OR exploration OR survey* OR interview* OR satisfact*).

Detailed presentation of the outcomes of the MMAT critical appraisal of the included studies

Screening questions performance: Not stating the research question clearly led to a downgrade in the quality of the study by Freeman et al., (2022). Research objectives appeared ambiguous as it remained unclear which specific qualitative data the authors intended to capture. This led to the judgement of the appropriateness of the data collected and the subsequent methodological quality criteria impossible. Therefore, the study was categorised as having ‘poor quality’ due to difficulty determining if it meets the MMAT criteria.

Sampling strategies and nonresponse bias: All studies used non-probability sampling, but rarely state a specific strategy (Stenvinkel et al., 2013; Saeed et al., 2017; Suresh et al., 2020; Chan and Soucisse., 2016). Only Gupta et al., (2019) explicitly reports using convenience sampling but did not justify their choice per se. Sampling strategies were deemed relevant to address the research question in all studies. However, the only study to provide adequate descriptions of both the target and sample populations was Gupta et al., (2019). Selection criteria are specified by only two out of the four available studies (Gupta et al., 2019; Saeed et al., 2017). Whereas respective sizes of the target population were provided only by Suresh et al., (2020) (n=118) and Chan and Soucisse., (2016) (n=162). The latter described the target population only in terms of professional specialisation. The original, 30-item survey had a response rate of approximately 32%. Subsequently the survey was reduced to 18-items in view of decreasing the participant perceived efforts required to complete it. Contrastingly to the anticipated

effects, the shorter version resulted in an even lower response rate of 20% (Chan and Soucisse., 2016). The disparity was unaddressed. The sample population consisted primarily of nephrologists (74%) followed by transplant surgeons (21.3%), with 4% (n=2) responses completed collectively by the centre's transplant group. The number of potential respondents within the 18 available centres was not provided. Therefore, there is a risk of underrepresentation or oversampling within the sample. Further, authors report multiple responses to have been received from the same centres which could introduce bias, especially if the responses were not independent. Exact proportion of these responses is not stated. Similarly, Suresh et al., (2020) reports low response rates (26%) and does not provide reasons for participant nonresponse, or comment on the differences between nonrespondents and the sample. Therefore, whether the samples in these studies are representative of the target population could not be established.

Saeed et al., (2017) had a response rate of 85% with nonresponse attributed to twelve individuals either not being interested in participation, meeting exclusion criteria, or being hospitalised at the time of the study. The authors did not report any follow-up procedures for non-responders or any attempts of statistical compensation such as imputation. Gupta et al, (2019) reports exclusion of seven responders (5%) in accord with the selection criteria and was the only study to have low risk of non-response bias. Overall, there is a lack of clearly identified population of interest, diverse participant recruitment strategies, sampling randomisation or efforts to compensate for high rates of nonresponse. Particularly studies by Stenvinkel et al., (2013), Suresh et al., (2020) and Chan and Soucisse., (2016) are deemed high-risk for sampling and nonresponse bias.

Data collection methods: All quantitative studies used surveys as tools for data collection. Gupta et al., (2019) reported carrying out structured, individual, in-person interviews, however, does not report on the data collected. All four provide survey instruments yet only Chan and Soucisse., (2016) provides detail on survey design, such as attempts to reduce perceived response burden and improve response rate. Items were categorised as reflective of each study respective research question. None of the studies, however, pre-tested questionnaires or carried out pilot studies to determine questionnaire validity prior to data collection. Further, only Saeed et al., (2017) tested reliability of survey items. The studies do not report on data consistency and completeness, as well as agreed procedures for handling missing values. Therefore, all quantitative studies but Saeed et al., (2017) were categorised as high risk for measurement error.

Statistical analysis: Only Saeed et al., (2017) reports extensively on the statistical methods employed including techniques for accessing reliability and analysis of binary, ordinal and categorical data. Authors provide p-values for determining significance. Gupta et al., (2019) mentions employing bivariate analysis and multivariate logistic regression to estimate associations between self-perceived

patient variables such as transplant eligibility, BMI, years of education and awareness of transplantation centre weight limit. Measure values are provided together with 95% confidence intervals. Authors provide p-values for determining significance. Chan and Soucisse., (2016) report using standard descriptive statistical methods, without outlining the specifics. Data is presented as percentages of responses. Suresh et al., (2020) and Stenvinkel et al. (2013) do not specify methods employed for data analysis. Data was expressed as percentages of responses, and medians accompanied by interquartile ranges. Overall, appropriateness of statistical analysis could only be judged adequately for Saeed et al., (2017).

Table S1. Study selection criteria.

	Inclusion	Exclusion
Population	Adults with obesity (BMI ≥ 30 kg/m²) and kidney failure (GFR < 15 mL/min/1.73m^2) undergoing pre-transplant dialysis<br><br>Clinicians are defined as healthcare professionals directly involved in treating patients with kidney failure regardless of their specialisation.	Paediatric populations<br><br>Patients with chronic kidney disease stages 1 - 4
Intervention	Obesity management interventions including lifestyle, pharmacological and surgical approaches. Lifestyle approaches including those exploring diet, physical activity, or behavioural strategies used in isolation or in combination.	Assessments of health-related quality of life related to kidney disease, rather than obesity.
Study type	Peer-reviewed qualitative and quantitative studies published in English in which patients or clinicians report on their experiences of obesity management in kidney failure.	Studies published as abstracts, conference, or workshop reports.
Outcomes	Patient- and clinician-reported perspectives and experiences (including but not limited to motivations, expectations, and challenges) on obesity management in kidney failure	Quantitative findings reflecting rate of weight loss, changes in clinical parameters, dialysis, and therapeutic schedules.

BMI, body mass index; GFR, glomerular filtration rate; mL/min/1.73m^2 milliliters per minute per 1.73 square metres; kg/m², kilograms per metre squared

Table S2. MMAT quality assessment for the seven included studies.

Study	Screening questions (For all study types)		Methodological quality criteria for Qualitative studies (Yes/No/Can't tell)					Overall Quality score
	Are there clear research questions?	Do the collected data allow to address the research questions?	Is the qualitative approach appropriate to answer the research question?	Are the qualitative data collection methods adequate to address the research question?	Are the findings adequately derived from the data?	Is the interpretation of results sufficiently substantiated by data?	Is there coherence between qualitative data sources, collection, analysis and interpretation?	
Harhay et al., 2023	Yes	Yes	Yes	Yes	Yes	Yes	Yes	High
Freeman et al., 2022	CT	CT	CT	CT	CT	CT	CT	Poor
**Study**	**Screening questions (For all study types)**		**Methodological quality criteria for Quantitative studies (Yes/No/Can't tell)**					**Overall Quality score**
	Are there clear research questions?	Do the collected data allow to address the research questions?	Is the sampling strategy relevant to address the research question?	Is the sample representative of the target population?	Are the measurements appropriate?	Is the risk of nonresponse bias low?	Is the statistical analysis appropriate to answer the research question?	
Stenvinkel et al., 2013	Yes	Yes	CT	No	Yes	No	CT	Poor
Saeed et al., 2017	Yes	Yes	Yes	Yes	Yes	No	Yes	Fair
Chan and Soucisse, 2016	Yes	Yes	Yes	CT	CT	No	CT	Poor
Suresh et al., 2021	Yes	Yes	Yes	CT	CT	No	CT	Poor
Gupta et al., 2019	Yes	Yes	Yes	Yes	Yes	Yes	C	Fair

CT, can't tell
